# Supplementary figures and images for: Mechanisms of thrombin-Induced myometrial contractions: Potential targets of progesterone
Source: PLoS One. 2020 May 4;15(5):e0231944. doi: 10.1371/journal.pone.0231944 (PMC7197857; doi:10.1371/journal.pone.0231944)

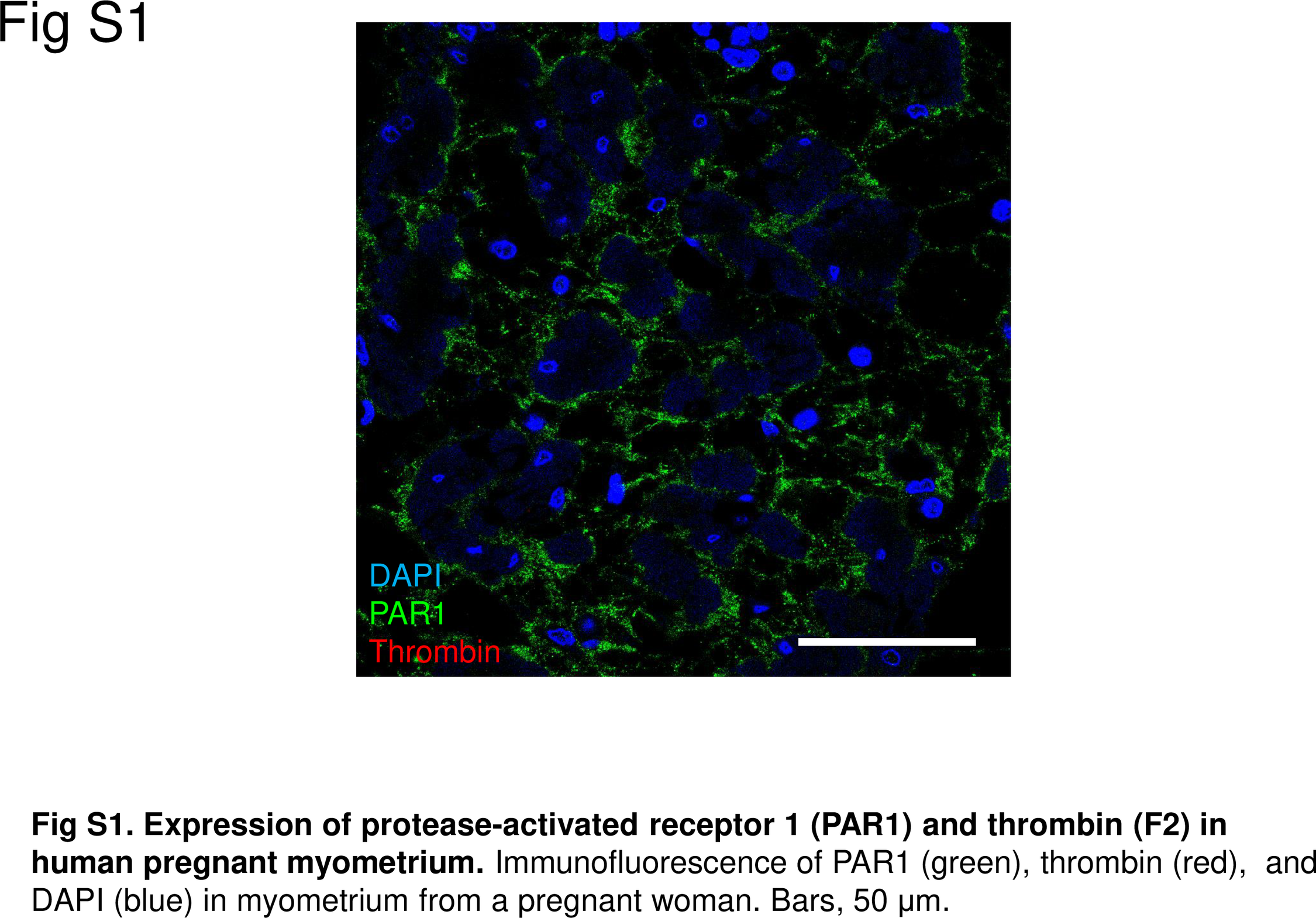

Supplement: S1 Fig — (TIF) [file pone.0231944.s001.tif]

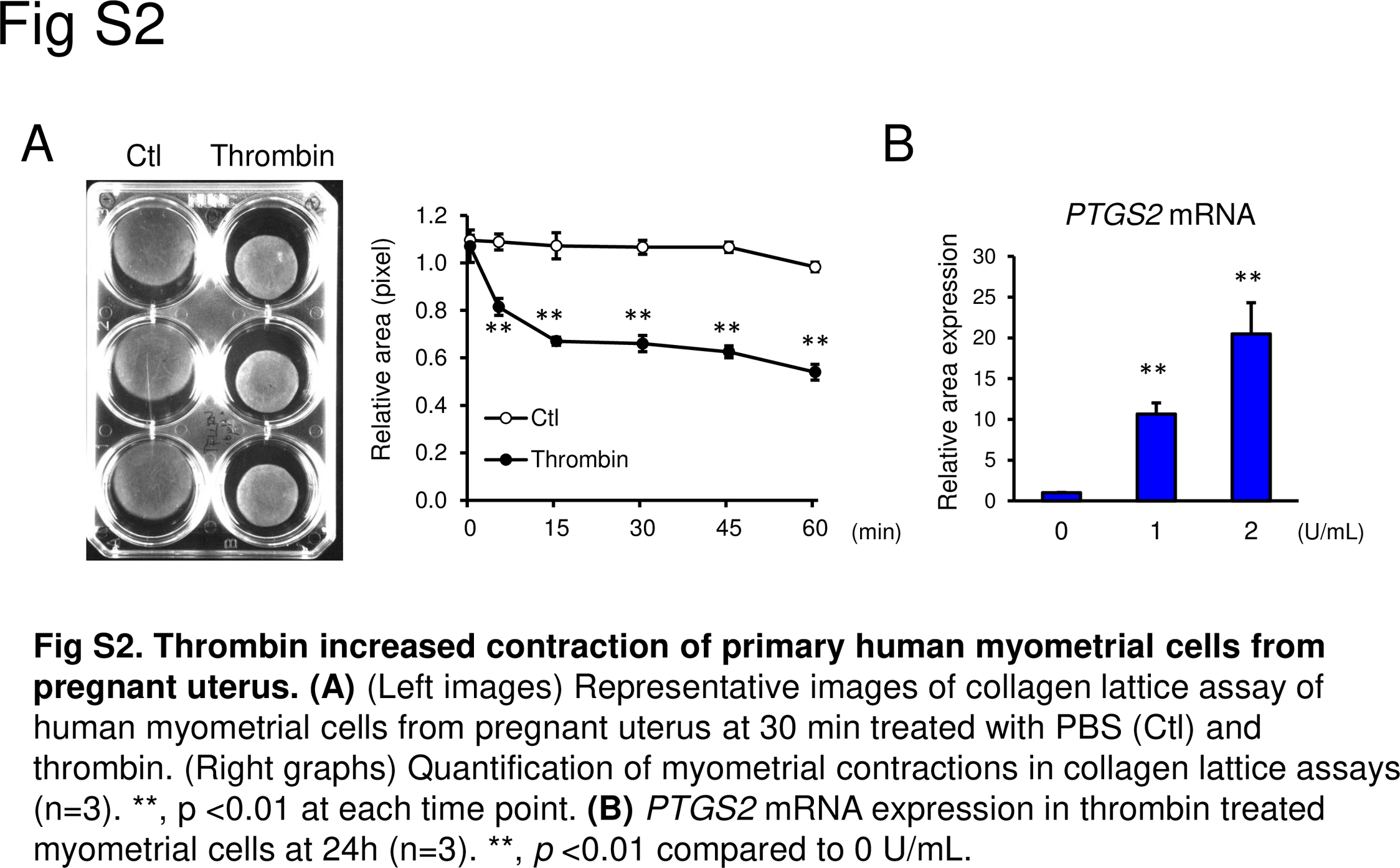

Supplement: S2 Fig — (TIF) [file pone.0231944.s002.tif]

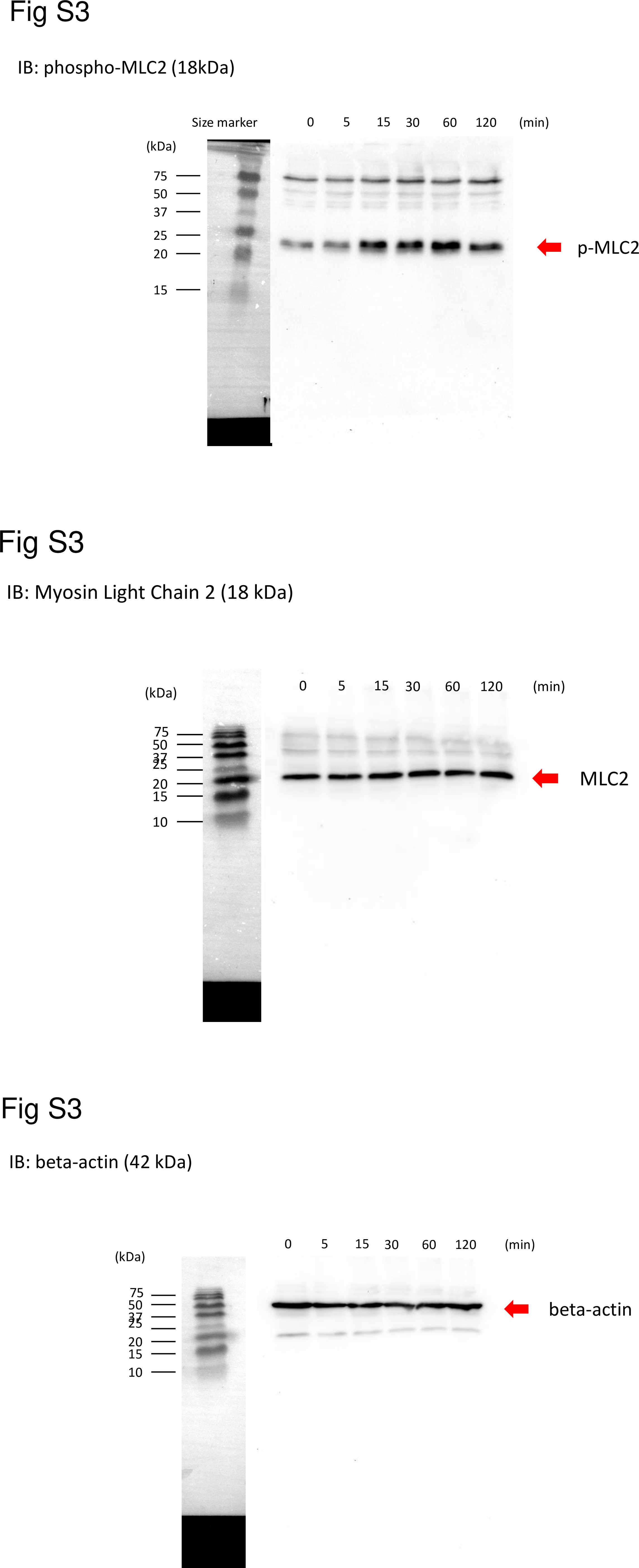

Supplement: S3 Fig — (TIF) [file pone.0231944.s003.tif]

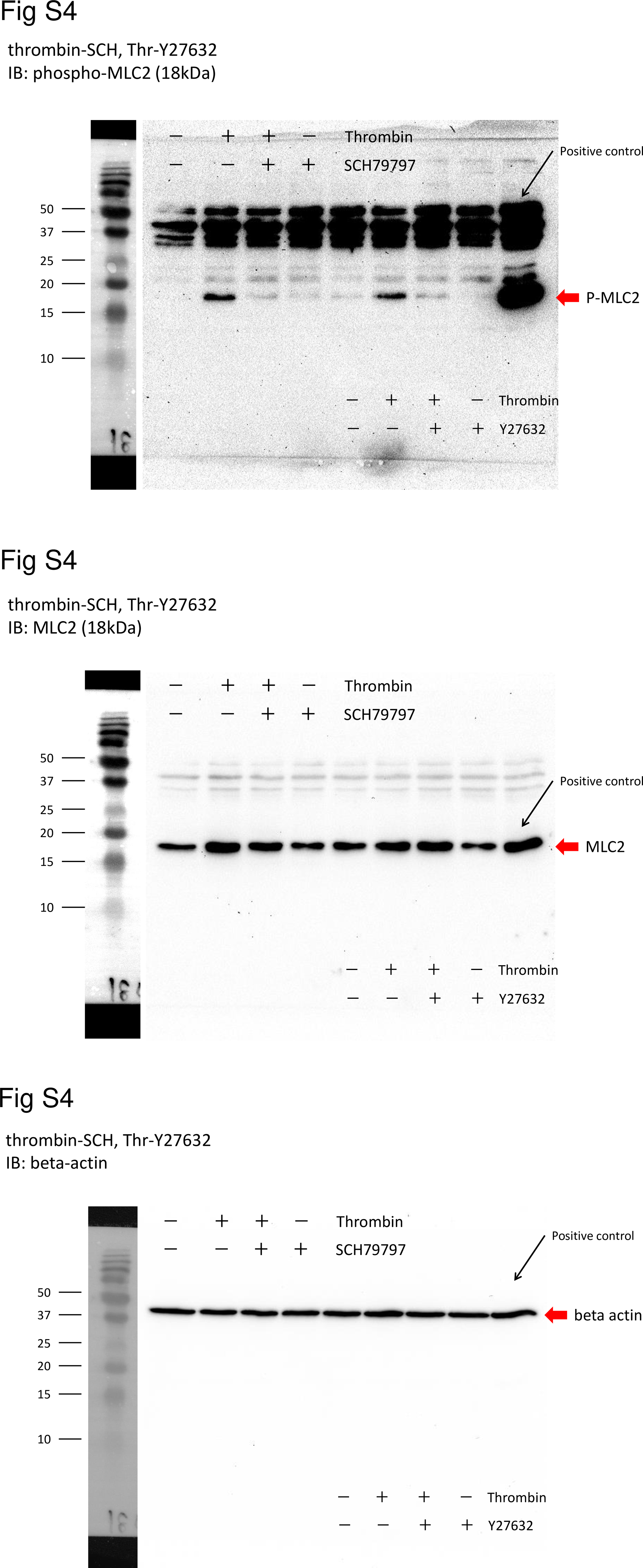

Supplement: S4 Fig — (TIF) [file pone.0231944.s004.tif]

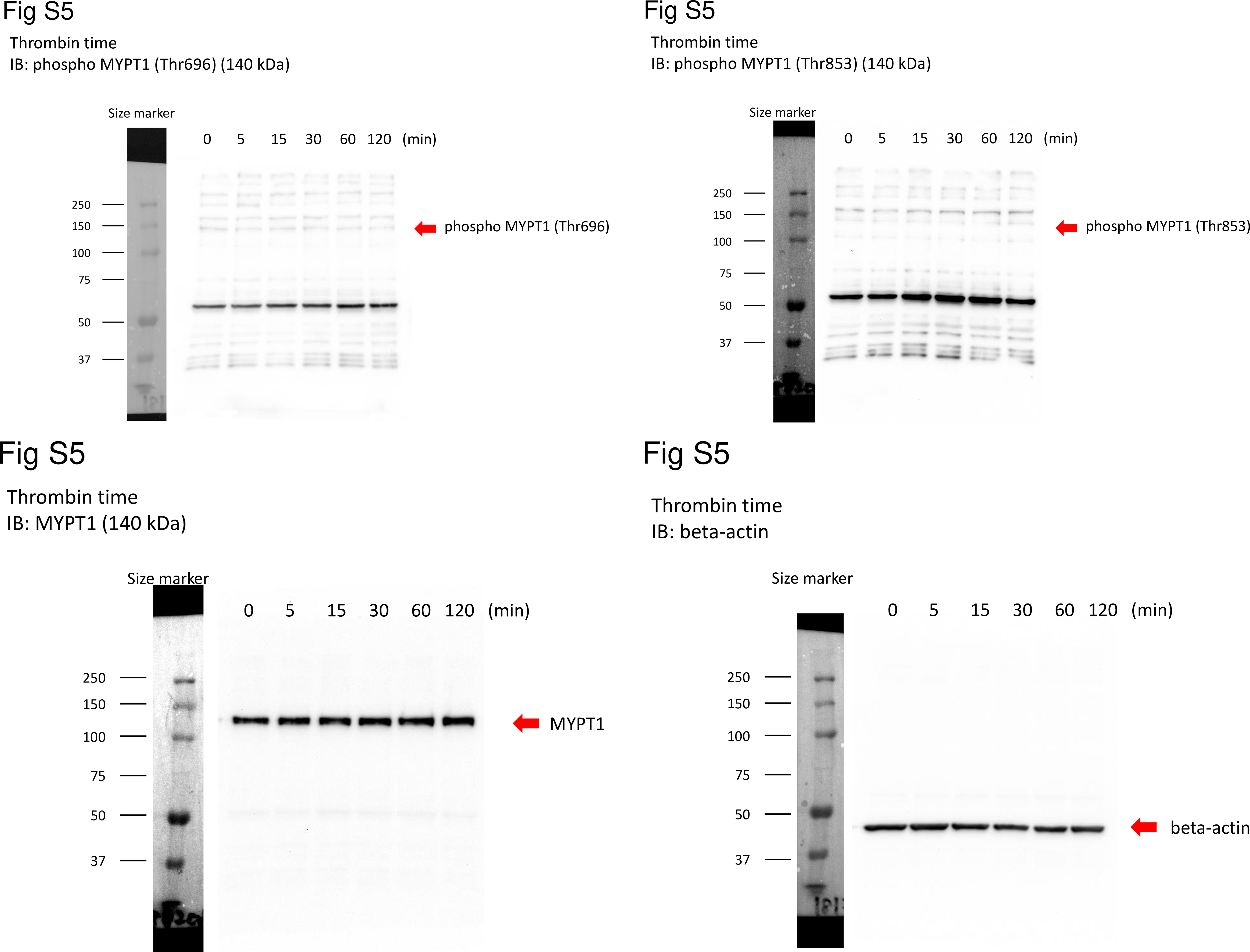

Supplement: S5 Fig — (TIF) [file pone.0231944.s005.tif]
